# Supplementary material for: Ex Vivo Murine Skin Model for B. burgdorferi Biofilm
Source: Antibiotics (Basel). 2020 Aug 19;9(9):528. doi: 10.3390/antibiotics9090528 (PMC7558507; doi:10.3390/antibiotics9090528)
Supplement: Supplementary file 1 [file antibiotics-09-00528-s001.pdf]

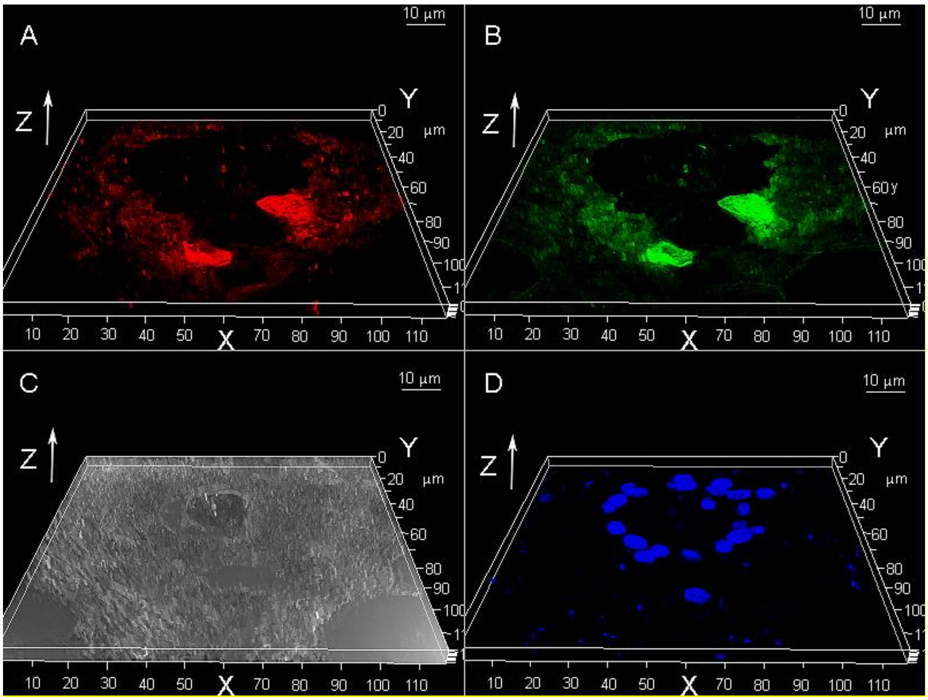


**Supplemental Figure S1: Three-dimensional analysis of biofilm form of *B.* *burgdorferi*** via confocal microscopy in infected biopsies inoculated with 1 × $10^7$ spirochetes and cultured in BSK-H 6% RS. Panel A shows biofilm marker alginate (red); Panel B illustrates *B. burgdorferi* (green), and Panel C shows DIC microscopy images and Panel D represents DAPI (blue) staining. 630x magnification. Scale bars show 10 µm.
